# Editorial: Polycystic ovary syndrome (PCOS): Mechanism and management

**DOI:** 10.3389/fendo.2022.1030353

**Published:** 2022-10-07

**Authors:** Yishu Wang, Peter Leung, Rong Li, Yanting Wu, Hefeng Huang

**Affiliations:** ^1^ International Peace Maternity and Child Health Hospital, School of Medicine, Shanghai Jiao Tong University, Shanghai, China; ^2^ Obstetrics and Gynecology Hospital, Institute of Reproduction and Development, Fudan University, Shanghai, China; ^3^ Shanghai Key Laboratory of Embryo Original Diseases, Shanghai, China; ^4^ Department of Obstetrics and Gynaecology, BC Children’s Hospital Research Institute, University of British Columbia, Vancouver, BC, Canada; ^5^ Centre for Reproductive Medicine, Department of Obstetrics and Gynecology, Peking University Third Hospital, Beijing, China; ^6^ National Clinical Research Centre for Obstetrics and Gynecology, Beijing, China; ^7^ Research Units of Embryo Original Diseases, Chinese Academy of Medical Sciences, Shanghai, China

**Keywords:** Polycystic Ovary Syndrome (PCOS), pathophysiology, diagnosis, prevention, obstetrical complications, offspring health, clinical management, microbiome

Polycystic ovary syndrome (PCOS) is the most common endocrine disorder in women of reproductive age with a reported prevalence ranging from 6% to 20% (1). In 2004, the economic impact of evaluating and providing care to persons with PCOS during the reproductive life span is $4.36 billion in the United States alone, even regardless of the cost of the increased risk of obstetrical and metabolic complications (2). The syndrome has severely impaired reproductive health and has already been a heavy health care-related economic burden. Unfortunately, our knowledge of PCOS remains limited. This Research Topic addresses a more nuanced picture of the pathophysiology and clinical investigations, as well as the management of PCOS.

PCOS has long been accepted as the major cause of anovulatory infertility and hirsutism, with an increased risk of developing metabolic abnormalities, type 2 diabetes mellitus (T2DM), obstetrical complications, mood disorders, cardiovascular and cerebrovascular events, venous thromboembolism, and endometrial and ovarian cancer (3). The condition is typified by androgen excess, ovulatory dysfunction, and polycystic ovarian morphology (PCOM), among which excessive androgen production by the ovaries is the key feature. Considering the heterogeneous presentation of PCOS, not all patients will have all three of these abnormalities, thus considerable debate exists regarding the criteria for the definition and the diagnosis of PCOS. In the diagnostic development of PCOS, three major sets of criteria have been accepted: (i) the criteria of the NIH and National Institute of Child Health and Human Disease (NIH–NICHD) in 1992 requires both hyperandrogenemia and ovulatory dysfunction; (ii) the Rotterdam criteria in 2003 requires any two of the three criteria: hyperandrogenemia, ovulatory dysfunction, and polycystic ovaries (4, 5); (iii) the AE-PCOS criteria by the Androgen Excess Society in 2006 underscores that the diagnosis is made only if they have hyperandrogenemia and either ovarian dysfunction, polycystic ovaries or both.

We asked the authors who participated in this Research Topic to consider the following questions: How to dive deep into the pathophysiology of PCOS? What are the new insights into metabolic dysfunctions, such as basal/glucose­ stimulated hyperinsulinemia and insulin resistance (IR), obesity, hyperandrogenism, and subfertility in PCOS? Among the repertoire of clinical management of PCOS, is there any exciting progress in pharmacological and lifestyle interventions? Is it possible to make a clinical diagnosis of PCOS through specific biomarkers? Can we effectively personalize assisted reproductive technology (ART) procedures for PCOS patients? How does PCOS influence maternal and offspring health? Why is it always associated with obstetrical complications including preeclampsia, very preterm birth (defined as <32 weeks of gestation), and gestational diabetes mellitus (GDM)? For the offspring of patients with PCOS, what symptoms are they inclined to display? What is the mechanistic role of the microbiome in PCOS? The result of this call is a relatively comprehensive collection of 33 articles regarding such aspects.

## Pathophysiology of PCOS

In reports of pioneering studies, the most consistent feature of PCOS is an elevated level of testosterone and/or androstenedione in serum (6). Ding et al. give a comprehensive review of the mutual role of IR and HA on PCOS development. Song et al. provide an experimental study in a rat model which demonstrates that androgen excess could damage mitochondrial ultrastructure by depressing the expression of NDUFB8 and ATP5j and thereby influence the function of granulosa cells (GCs) in PCOS. Furthermore, the risk of epilepsy and antiseizure medications on PCOS through the HPO axis is reviewed by Li et al.

Jiang et al. reveal that a higher expression of ANGPTL4 in GCs might be associated with glucose and lipid metabolic disorders in PCOS. Differently expressed elements of store-operated Ca^2+^ entry (SOCE) are analyzed in the work by Song et al. and are proved to contribute to the dysfunction of ovarian GCs and hormonal changes. Deng et al. conduct a whole genome transcriptomic sequencing and find DLGAP5 as a candidate gene for PCOS.

Immune balance and immune microenvironment may play a significant role in the infertility of PCOS patients. Liu et al. show that IL-15 affects the inflammation state, steroidogenesis, and survival of GCs. Ding et al. indicate that adipose tissue-derived extracellular vesicles-miR-26b promote GCs apoptosis. To supplement, Zhou et al. review the seminal features and therapeutic potential of extracellular vesicles in PCOS. Finally, Gu et al. have an extensive discussion on the relationship between the microbiome and sexual hormones, immune homeostasis as well as insulin resistance.

## Screening and prevention of PCOS

Since the expense of the diagnostic evaluation accounted for only a minor part of the total costs, more liberal screening for PCOS appears to be a cost-effective strategy. Given the probable multifactorial cause of PCOS, a specific plan for early risk prediction of diagnosis is not yet possible. Hence, further studies of the early biomarkers of PCOS and early intervention of at-risk adolescents are sorely needed. He et al. provide an update that increased apolipoprotein B/A1 ratio is associated with worse metabolic syndrome components, hyperandrogenemia, IR, and elevated liver enzymes. The new aspect of excessive visceral adipose tissue mass is analyzed in the contribution by Zhang et al., demonstrating it is this characteristic but not other fat compartments that can exacerbate the risk of hyperuricemia in PCOS. Yang et al. describe the positive correlation between neck circumference and serum uric acid levels. The increased risk factor of PCOS is examined in the contribution of Chen et al., who defined IL-17, SDF1a, SCGFb, and IL-4 as potential biomarkers for PCOS. With the rapid development of artificial intelligence, Lv et al. propose an automated deep learning algorithm for exploring the potential of scleral changes in PCOS detection.

## Maternal and offspring health of PCOS

Patients with PCOS are at risk of experiencing obstetrical complications including pre­eclampsia, very preterm birth, and GDM (7). Their offspring have a significantly higher risk of large for gestational age, meconium aspiration syndrome, and low Apgar scores at 5 minutes. Definitely, PCOS is a threat to maternal and child health.

The issue of cumulative live birth rate (CLBR) in PCOS is dealt with by two articles. Mai et al. demonstrate that PCOS exhibited higher CLBR and better ovarian reserve and response. When it comes to the independent variables for determining CLBR of aged patients with PCOS, this view is counter-argued by Guan et al., with a retrospective cohort study presenting significantly decreased CLBR for females of advanced reproductive age up to 37. Compared to regular menstruation or oligomenorrhea, a higher overall incidence of adverse pregnancy outcomes in PCOS patients with amenorrhea is presented by Yu et al. In addition, Du et al. point out that preterm birth in PCOS is associated with a BMI≥24 kg/m2 plus serum AMH>6.45 ng/ml. According to Jiang et al., advanced age, obesity, total cholesterol, triglycerides, and insulin resistance (IR) are all independent risk factors for a lower chance of achieving a live birth.

Two articles, by Zhang et al. and Jiang et al. respectively, focus on the health of PCOS offspring and broaden our understanding of PCOS offsprings’ cardiometabolic status and autistic traits. Xie et al. have generated letrozole-induced PCOS-IR rat models and treated them with metformin. In this article, they interpret that metformin might improve obesity, hyperinsulinemia, and IR in female offspring.

## Management of PCOS

As the primary treatment of metabolic dysfunction in PCOS, lifestyle interventions prevent progression to T2DM and lower cardiovascular risk as well as improve ovulation in 40–50% of patients with PCOS (8, 9). Gu et al. review the current evidence of the role of lifestyle modifications in PCOS, including diet modifications, exercise modifications, sleep modifications, mood modifications, and weight modifications. A meta-analysis by Shang et al., involving 20 RCTs with 1113 participants, depicts that diet intervention significantly improves fertility outcomes, reproductive endocrine, and clinical hyperandrogenism. Shen et al. submit another meta-analysis, which regards tea supplements as adjuvant therapy for improvement in body weight, fasting blood glucose, and insulin. Through remodeling gut microbiota, Wang et al. show that a high-fiber diet could alleviate chronic metabolic inflammation, reproductive function, and brain-gut peptides secretion in their study.

Functional abnormalities of adipose tissue do exist in PCOS patients, mainly manifesting as IR and inflammation (10). As anticipated from previous reports, human PCOS patients had lower brown adipose tissue (BAT) activity (11). The BAT transplantation experiment was conducted by Yao et al., which underscores the role of metabolome changes for BAT transplantation in improving reproductive and metabolic phenotypes in PCOS. Furthermore, Ye et al. pinpoint that cold treatment could also improve ovulation and hormone disorders *via* activating endogenous BAT.

Sleeve gastrectomy (SG) is a popular bariatric surgical procedure. However, its suitability and potential mechanisms in PCOS remain ambiguous. Lin et al. provide a novel perspective on the regulation of microbial taxa and SCFA content after SG, which might explain the mechanism of the amelioration of PCOS-related reproductive and metabolic disorders.

Due to the poor quality of oocytes retrieved from patients with PCOS, IVF treatment is always accompanied by lower-quality embryos with a low implantation rate (12). Chen et al. compare the implantation rate, clinical pregnancy rate, and live birth rate between progestin-primed ovarian stimulation and the short protocol. The former shows significantly more positive results.

## Concluding remarks

Although a substantial fraction of information has been added to the existing pool of knowledge of PCOS in the past decades, much remains to be elucidated. We sincerely thank all contributors and reviewers for their support in putting this timely collection of articles together and hope that the readers will find useful answers to their questions.

## Author contributions

YiW drafted this editorial. PL, RL, YaW and HH revised and approved the final submitted version.

## Funding

This work is supported by the National Key Research and Development Program of China (2021YFC2700701), the National Natural Science Foundation of China (82088102), CAMS Innovation Fund for Medical Sciences (2019-I2M-5-064), Collaborative Innovation Program of Shanghai Municipal Health Commission (2020CXJQ01), Clinical Research Plan of SHDC (SHDC2020CR1008A) and Shanghai Frontiers Science Research Base of Reproduction and Development.

## Conflict of interest

The authors declare that the research was conducted in the absence of any commercial or financial relationships that could be construed as a potential conflict of interest.

## Publisher’s note

All claims expressed in this article are solely those of the authors and do not necessarily represent those of their affiliated organizations, or those of the publisher, the editors and the reviewers. Any product that may be evaluated in this article, or claim that may be made by its manufacturer, is not guaranteed or endorsed by the publisher.
